# Kollaborationen bei kinderanästhesiologischen Publikationen im D-A-CH-Raum

**DOI:** 10.1007/s00101-024-01459-5

**Published:** 2024-09-24

**Authors:** Alexander Dejaco, M. Nemeth, A. Sablewski, J. Rosenberger, C. Miller

**Affiliations:** 1 Klinik für Anästhesiologie, Universitätsklinikum Regensburg, Franz-Josef-Strauß-Allee 11, 93053 Regensburg, Deutschland; 2 Klinik für Anästhesiologie, Universitätsmedizin Göttingen, Göttingen, Deutschland; 3 Klinik für Anästhesiologie und Operative Intensivmedizin, Universitätsklinikum Schleswig-Holstein, Campus Kiel, Kiel, Deutschland; 4 Neurochirurgische Intensivmedizin, Universitätsklinik für Neurochirurgie, Medizinische Universität Innsbruck, Innsbruck, Österreich; 5 Abteilung für Anästhesiologie, Orthopädische Kinderklinik Aschau im Chiemgau, Aschau im Chiemgau, Deutschland

**Keywords:** Szientometrie, Bibliometrie, Zusammenarbeit, Publikationsaktivität, Vernetzung, Scientometrics, Bibliometrics, Collaborations, Publication activity, Networking

## Abstract

**Hintergrund:**

Kollaborationen sind ein wesentlicher Baustein wissenschaftlichen Arbeitens und speziell für Bereiche wie die Kinderanästhesiologie besonders bedeutsam, wo ein geringes Evidenzlevel besteht. Eine kürzlich durchgeführte szientometrische Analyse ergab eine geografische Diversifizierung der Publikationsaktivität in der Kinderanästhesie innerhalb der letzten 2 Jahrzehnte, begleitet von einem Anstieg internationaler Kooperationen.

**Ziele:**

Vor dem Hintergrund der Hypothese eines ähnlichen Wachstums der Aktivität und Dynamik von Veröffentlichungen in der Kinderanästhesie bestand das Ziel dieser szientometrischen Studie darin, die Publikationsaktivität und Kooperationsgewohnheiten in der Kinderanästhesieforschung in Deutschland, Österreich und der Schweiz (D-A-CH) zu analysieren.

**Methode:**

Diese Sekundäranalyse schloss alle kinderanästhesiologischen Publikationen mit Zugehörigkeit aus dem D‑A-CH-Raum zwischen 2001 und 2020 aus PubMed und Web of Science ein. Publikationen wurden anhand der Korrespondenzadresse zugeordnet. Primärer Endpunkt war die Publikationsaktivität und -dynamik, dargestellt anhand der Anzahl der Veröffentlichungen und den jeweiligen Wachstumsraten. Sekundäre Endpunkte waren die Anteile an Kollaborationen auf staatlicher und institutioneller Ebene.

**Ergebnisse:**

Insgesamt wurden 3406 kinderanästhesiologische Publikationen mit Beteiligung aus dem D‑A-CH-Raum identifiziert, davon 2807 (82,4 %) mit Korrespondenzadresse. Die durchschnittliche jährliche Wachstumsrate an Publikationen mit Korrespondenzadresse lag für den D‑A-CH-Raum bei + 2,9 % und bei Kollaborationen + 7,7 %. Kollaborationen fanden überwiegend zwischen Institutionen innerhalb des D‑A-CH-Raums statt, aber Schweizer Institutionen hatten einen höheren Anteil an internationalen Kollaborationen.

**Diskussion:**

Die Aktivität bei kinderanästhesiologischen Publikationen aus dem D‑A-CH-Raum nahm in den letzten beiden Dekaden stetig zu, wobei Kollaborationen eine überproportionale Zunahme verzeichneten. Es bleibt zu wünschen, dass sich mit der wachsenden Bedeutung von Kollaborationen auch die Evidenzlage in der Kinderanästhesie weiter verbessert.

## Abstract (en)

**Background:**

Collaborations are an essential element of scientific activity and particularly important in fields such as pediatric anesthesiology, where the evidence base in general is relatively limited. A recent scientometric analysis revealed a geographic diversification of publication activity in pediatric anesthesiology within the last two decades, accompanied by a surge in international collaborations.

**Objectives:**

Given the hypothesis of a similar growth in the activity and dynamics of publications in pediatric anesthesiology, the objective of this scientometric study was to analyze the publication activity and collaboration habits in research in pediatric anesthesiology from Germany, Austria and Switzerland (D-A-CH).

**Method:**

This secondary analysis identified all publications on pediatric anesthesiology with an affiliation from the D‑A-CH countries between 2001 and 2020 from PubMed and Web of Science. The query parameters included the timeframe 2001–2020, authors’ affiliations tied to anesthesiology departments (using various forms of the term “anesthesia”), and the mention of pediatric interest in titles or abstracts. The data underwent standardization to account for linguistic variations. The publications were assigned to a state, city and institution based on the correspondence address, to a year based on the publication date and to a source based on the journal. The primary endpoint was publication activity and dynamics, represented by the number of publications and the respective growth rates (calculated as the linear regression slope). Secondary endpoints included the share of collaborations within and outside the D‑A-CH region (at the country and institutional level), the distribution of publication activity and the most prominent sources of publications.

**Results:**

Between 2001 and 2020 a total of 3406 publications on pediatric anesthesiology involving authors from the D‑A-CH countries were identified. Of these 2807 (82.4%) had a correspondence address in D‑A-CH. The average annual growth rate of publications with a correspondence address was + 2.9% for the D‑A-CH countries and + 7.7% for publications with collaborations. The number of publications in which an institution from D‑A-CH was named as a coauthor from a correspondence address outside D‑A-CH also increased by an average of 7.4% per year during the study period. The majority of collaborations occurred between institutions within the D‑A-CH region, although Swiss institutions exhibited a much higher proportion of collaborations outside the region. Of all publications with a correspondence address 90% originated from 46 cities. The most prominent source was *Die Anästhesiologie* for publications from Germany, and *Pediatric Anesthesia* for publications from Austria and Switzerland.

**Conclusion:**

The number of publications in pediatric anesthesiology from the D‑A-CH countries has increased over the past two decades, accompanied by a surge in collaborations. It is hoped that increased collaboration will contribute to a higher level of evidence in pediatric anesthesiology care.

Kollaborationen spielen in der medizinischen Wissenschaft eine zunehmend größere Rolle. Dies ist v. a. für Spezialisierungen wie die Kinderanästhesiologie wichtig, um bestehende Evidenzlücken zu schließen. Anhand dieser szientometrischen Analyse werden die Publikationsaktivität und -dynamik sowie der nationale und internationale Vernetzungsgrad bei kinderanästhesiologischer Forschung in Deutschland, Österreich und der Schweiz dargestellt.

## Hintergrund und Fragestellung

Wissenschaftliche Arbeit ist essenziell zur Evidenzgenerierung, für die Entwicklung von Leitlinien sowie für die kontinuierliche Aus- und Weiterbildung und trägt damit entscheidend zur Weiterentwicklung eines Fachgebietes bei [5]. Durch die Veröffentlichung wissenschaftlicher Arbeiten in Fachzeitschriften werden Forschungsergebnisse und andere für das Fachgebiet relevante Inhalte wie aktuelle Therapieempfehlungen kommuniziert und verbreitet. Kollaborationen zwischen Institutionen sind hierfür ein wichtiger Impulsgeber [1].

Dies trifft auch auf die Kinderanästhesiologie zu, wo in vielen Bereichen ein geringes Evidenzlevel besteht. Diese Evidenzlücken zu schließen, gelingt zunehmend durch internationale Kollaborationen, wie beispielsweise bei den rezent publizierten, großen multizentrischen Studien APRICOT oder NECTARINE [4, 7]. Die durch die Fachgesellschaften wie den Wissenschaftlichen Arbeitskreis Kinderanästhesie der Deutschen Gesellschaft für Anästhesiologie und Intensivmedizin (DGAI), die European Society of Paediatric Anaesthesiology oder die World Federation of Societies of Anesthesiologists etablierten Vernetzungen spielen dabei heute eine wesentliche Rolle [12].

Dies wird auch durch eine kürzlich veröffentlichte szientometrische Analyse von weltweiten Publikationen in der Kinderanästhesiologie der letzten zwei Dekaden unterstützt: Bei einem generellen Anstieg der Publikationsaktivität – analog zu einem Anstieg der Anzahl der Publikationen im gesamten Fachgebiet der Anästhesiologie [2] – war v. a. das überproportionale Wachstum an internationalen Kollaborationen in der Kinderanästhesiologie auffällig [9]. Wie sich die Publikationsaktivität und der Vernetzungsgrad in der Kinderanästhesiologie innerhalb von Deutschland, Österreich und der Schweiz (D-A-CH) entwickelt haben, wo zuletzt von einem Rückgang der Originalarbeiten in der Anästhesiologie berichtet wurde [8], ist bislang unbekannt.

Mit der Hypothese, dass die Zahl der kinderanästhesiologischen Publikationen im D‑A-CH-Raum zugenommen und sich v. a. das Kollaborationsverhalten über die letzten beiden Jahrzehnte deutlich ausgeweitet hat, war es das Ziel dieser szientometrischen Arbeit, die Publikationsaktivität und -dynamik sowie den nationalen und internationalen Vernetzungsgrad zu analysieren.

## Methoden

Wir führten eine szientometrische Analyse durch, welche kinderbezogene Publikationen mit anästhesiologischer Autorenschaft aus den D‑A-CH-Staaten der Jahre 2001 bis 2020 untersuchte. Erfasst wurden alle Artikeltypen von Publikationen in gelisteten Fachzeitschriften. Das angewandte Abfrageschema bedingt die Inklusion aller 5 Säulen der Anästhesiologie, wobei sämtliche inkludierten Publikationen unter dem Oberbegriff „Publikationen in der Kinderanästhesiologie“ subsumiert wurden. Da keine persönlichen oder sensiblen Daten verarbeitet wurden, war weder ein Ethikvotum noch eine Studienregistrierung erforderlich.

### Datengrundlage

Als Grundlage diente die Datenbank und anschließende Datenverarbeitung aus der globalen Publikationsanalyse der Kinderanästhesiologie von Miller et al. [9], welche Abfragen von Web of Science und PubMed mit Stichtag 04.10.2021 kombinierte. Diese Quellen gelten als die beiden bedeutendsten Publikationsdatenbanken im medizinischen Bereich [6], wobei Web of Science aufgrund vollständigerer Datensätze als primäre Datenquelle genutzt und fehlende Daten mittels PubMed ergänzt wurden [9].

Um kinderanästhesiologische Publikationen bestmöglich zu identifizieren, kombinierte die Suchabfrage 2 Parameter: Einerseits wurde die Zugehörigkeit mindestens einer Autorenschaft zu einer anästhesiologischen Institution erfasst, indem der Stamm des Wortes Anästhesiologie im Zugehörigkeitsfeld („affiliation field“) abgefragt wurde (beispielsweise durch „anaest*“, „anest*“ plus sprachbedingt mögliche Präfixe wie „Kinder-*“) [8]. Andererseits wurden kinderbezogene Publikationen durch Stichworte wie „Kind*“, „infant*“, „pediat*“ oder „child*“ im Titel oder im Abstract abgefragt. Verschiedenste sprachliche Variationen wurden berücksichtigt. Zur Beschränkung auf die letzten zwei Dekaden wurden die Publikationen nach ihrem Erscheinungsjahr zwischen 2001 und 2020 gefiltert. Die genaue Abfragesystematik ist bei Miller et al. einzusehen [9].

### Datenverarbeitung

Anhand der Korrespondenzadresse im Zugehörigkeitsfeld wurde jeder Publikation eine Institution, eine Stadt (eine Stadt kann mehrere publizierende Institutionen beinhalten) und ein Staat (D, A, oder CH) zugewiesen. War keine primäre Korrespondenz unter den Autorenschaften genannt (was insbesondere bei Daten von PubMed vor 2014 vorkommt [10]), wurde die Anschrift der Erstautorenschaft als Korrespondenzadresse verwendet. Sprachliche Varianten von Institutionsnamen (z. B. Hannover Medical School und Medizinische Hochschule Hannover) oder Städtenamen (z. B. Cologne und Köln) wurden entsprechend homogenisiert.

War bei einer Publikation mehr als eine Institution genannt, wurde die Publikation bei jeder nicht als Korrespondenz genannten Institution als Co-Autorenschaft gezählt. Dadurch ergab sich im Fall von Mehrfachnennungen zu jeder Korrespondenzadresse eine Liste an Kollaborationspartnerschaften. Hierfür wurde eine vollständige Zählweise verwendet.

Zudem wurden die Publikationen dem jeweiligen Erscheinungsjahr sowie der Fachzeitschrift zugeordnet. Auch bei den Fachzeitschriften erfolgte eine Homogenisierung bei Namensänderungen im Untersuchungszeitraum (z. B. wurde *Der Anaesthesist* zu *Die Anaesthesiologie*).

### Studienendpunkte

Primärer Endpunkt war die Erhebung der Publikationsaktivität und -dynamik in der Kinderanästhesiologie im D‑A-CH-Raum zwischen 2001 und 2020, dargestellt anhand der Anzahl der Veröffentlichungen mit Korrespondenzautorenschaft und den jeweiligen Wachstumsraten der drei Staaten. Sekundäre Endpunkte waren (i) der Anteil an Kollaborationen inner- und außerhalb des D‑A-CH-Raums, (ii) die Verteilung der Publikationsaktivität und der Anteil an Kollaborationen auf institutioneller Ebene innerhalb des D‑A-CH-Raums und (iii) die bedeutendsten Fachzeitschriften für Publikationen aus dem D‑A-CH-Raum.

### Statistik

Numerische Daten wurden als absolute Zahlen oder Prozentsätze angegeben und auf eine Kommastelle gerundet. Die Wachstumsrate über einen Zeitraum *t *wurde berechnet als $\left(\frac{\textit{Anzahl Erstjahr}}{\textit{Anzahl Letztjahr}}\right)^{\frac{1}{t}}-1$, wobei für den Gesamtzeitraum von 2001 bis 2020 t = 20 Jahre galt. Um die individuelle durchschnittliche Wachstumsrate einzelner Institutionen oder Staaten zu bestimmen, wurde eine lineare Regression der jeweiligen jährlichen Publikationszahlen durchgeführt und die Steigung der Regressionsgeraden als durchschnittliche Wachstumsrate für den Gesamtzeitraum verwendet.

Zu Datenverarbeitung und -auswertung setzten wir LibreOffice Calc 7.6.4 sowie JupyterLab 4.0.11 mit Python 3.11 ein. Für Illustrationen und Statistikauswertungen wurden zudem die Python-Bibliotheken matplotlib 3.8.1, GeoPandas 0.14.2 und scikit-learn 1.3 verwendet sowie folium 0.16 zur Erstellung der interaktiven webbasierten Landkarte.

## Ergebnisse

**Abb. 1:**
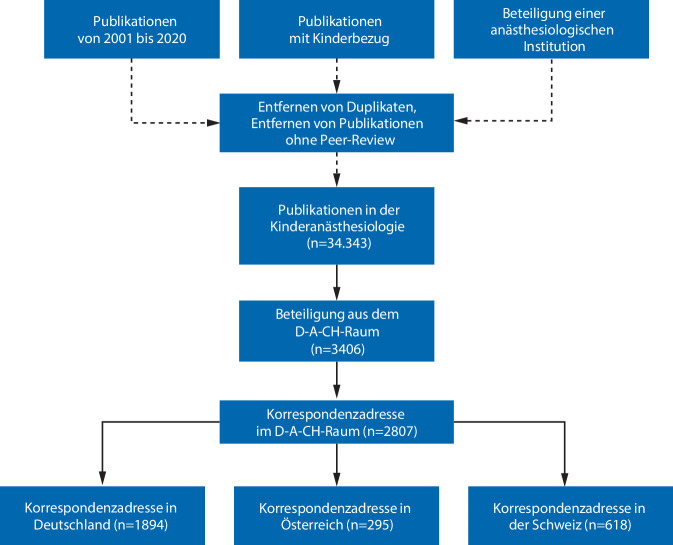
Flussdiagramm der Datenverarbeitung. *Gestrichelt* Datengrundlage aus Miller et al. 2024 [9]

**Abb. 2:**
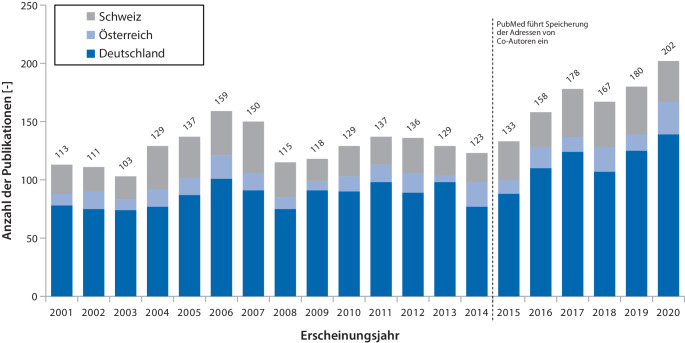
Anzahl der kinderanästhesiologischen Publikationen mit Korrespondenzadresse aus Deutschland (*blau*), Österreich (*hellblau*) und der Schweiz (*grau*) von 2001 bis 2020

Zwischen 2001 und 2020 wurden insgesamt 3406 kinderanästhesiologische Publikationen mit Beteiligung aus dem D‑A-CH-Raum identifiziert (Abb. 1). Davon wiesen 2807 (82,4 %) Publikationen eine Korrespondenzadresse im D‑A-CH-Raum auf. Bei 1894 lag diese in Deutschland, bei 295 in Österreich und bei 618 in der Schweiz (Abb. 2). Die durchschnittliche jährliche Wachstumsrate der Publikationen mit Korrespondenzadresse lag für den D‑A-CH-Raum bei + 2,9 %. Für Deutschland lag sie bei + 2,9 %, für Österreich bei + 5,3 %, und für die Schweiz bei + 1,7 %.

Der Anteil jener Publikationen, bei denen eine Institution aus dem D‑A-CH-Raum eine Co-Autorenschaft zu einer Institution mit Korrespondenzadresse außerhalb des D‑A-CH-Raums hatte, nahm im Untersuchungszeitraum um durchschnittlich 7,4 % pro Jahr zu (von 5,8 % 2001 auf 24,3 % 2020).

### Kollaborationen auf staatlicher Ebene

**Abb. 3:**
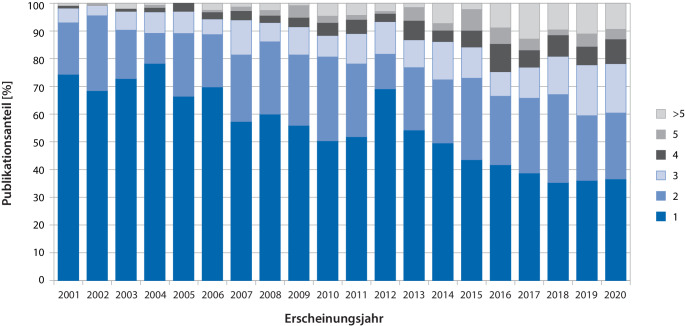
Prozentuale Verteilung der kinderanästhesiologischen Publikationen je nach Anzahl der Institutionen im „affiliation field“ (*1*, *2*, *3*, *4*, *5,* *>* *5*) mit Korrespondenzadresse aus Deutschland, Österreich oder der Schweiz von 2001 bis 2020

Von den 2807 Publikationen mit Korrespondenzadresse im D‑A-CH-Raum hatten 1296 (46,2 %) mindestens eine weitere Kollaborationspartnerschaft. Die jeweilige Anzahl an Publikationen mit zwei, drei, vier, fünf und mehr als fünf Kollaborationspartnerschaften nahm zwischen 2001 und 2020 zu (Abb. 3). Die durchschnittliche jährliche Wachstumsrate der Publikationen mit mindestens einer weiteren Kollaborationspartnerschaft betrug + 7,7 %.

Mit Korrespondenzadresse aus Deutschland waren 908 (47,9 % von 1894) der Publikationen Kollaborationen. Davon waren 627 (69,0 %) ausschließlich innerhalb des D‑A-CH-Raums, 173 (19,1 %) gemischt mit Kollaborationspartnerschaften inner- und außerhalb und 108 (11,9 %) ausschließlich außerhalb des D‑A-CH-Raums.

Mit Korrespondenzadresse aus Österreich waren 133 (45,1 % von 295) der Publikationen Kollaborationen. Davon waren 80 (60,1 %) ausschließlich innerhalb des D‑A-CH-Raums, 16 (12,0 %) gemischt inner- und außerhalb und 37 (27,8 %) ausschließlich außerhalb.

Mit Korrespondenzadresse aus der Schweiz waren 255 (41,3 % von 618) der Publikationen Kollaborationen. Davon waren 101 (39,6 %) ausschließlich innerhalb des D‑A-CH-Raums, 38 (14,9 %) gemischt inner- und außerhalb und 116 (45,5 %) ausschließlich außerhalb.

### Institutionelle Ebene

**Abb. 4:**
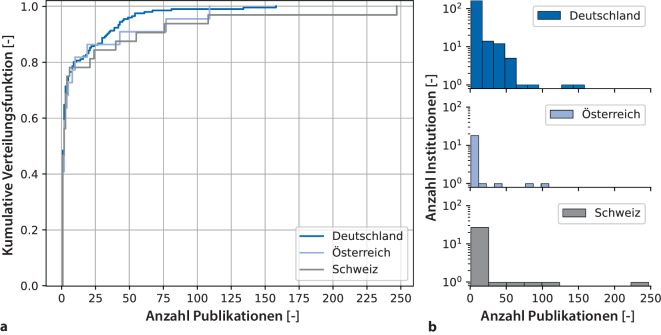
**a** Kumulative Aktivität mit Korrespondenzadresse von kinderanästhesiologischen Publikationen, differenziert nach Institutionen aus Deutschland, Österreich und der Schweiz von 2001 bis 2020. Zur Erläuterung: Ein Punkt auf der Kurve ergibt den Anteil y der Institutionen, die ≤ x Publikationen veröffentlicht haben. **b** Histogramme der Publikationszahlen, differenziert nach Institutionen in halblogarithmischer Darstellung

Die 2807 Publikationen mit Korrespondenzadresse im D‑A-CH-Raum wurden von 251 Institutionen aus 163 Städten veröffentlicht. Davon stammen 197 Institutionen aus Deutschland, 22 aus Österreich und 32 aus der Schweiz. Bezüglich der Verteilung stammen über 90 % aller Publikationen mit Korrespondenzadresse von 68 Institutionen aus 46 Städten (Abb. 4). Diese 46 haben jeweils mindestens 10 Publikationen zwischen 2001 und 2020 veröffentlicht.

Co-Autorenschaften inklusive waren 370 verschiedene Institutionen aus dem D‑A-CH-Raum an kinderanästhesiologischen Publikationen beteiligt; 295 aus Deutschland, 31 aus Österreich und 44 aus der Schweiz.

### Kollaborationen auf institutioneller Ebene

Publikationen aus der Stadt Genf hatten mit 179 die meisten Kollaborationspartnerschaften, während Publikationen aus der Stadt Essen mit 78,3 % den höchsten Anteil an Publikationen mit mindestens einer Kollaborationspartnerschaft hatten (Tab. 1).

**Tab. 1:** Übersicht der jeweiligen „Top-Ten“-Städte (mit ≥ 10 Publikationen zwischen 2001 und 2020) bezüglich Kollaborationspartnerschaften und anteiligen Publikationen mit Kollaborationspartnerschaften sowie Anteile der Kollaborationen des D‑A-CH-Raums (innerhalb, außerhalb und gemischt). Letztere Prozentangaben beziehen sich relativ auf jene Publikationen mit mind. 1 Kollaboration

Anzahl der Kollaborationspartnerschaften nach Städten		Anzahl und Anteil jener Publikationen mit mindestens einer Kollaborationspartnerschaft			Publikationen mit Kollaborationen ausschließlich innerhalb des D‑A-CH-Raums			Publikationen mit Kollaborationen ausschließlich außerhalb des D‑A-CH-Raums			Publikationen mit Kollaborationen gemischt inner- und außerhalb des D‑A-CH-Raums		
Stadt	*n*	Stadt	*n*	%	Stadt	*n*	%	Stadt	*n*	%	Stadt	*n*	%
Genf	179	Essen	18	78,3	Homburg	3	100	Basel	25	64,1	Stuttgart	1	50
Köln	119	Bochum	10	76,9	Klagenfurt	13	100	Genf	39	60,0	Mainz	4	44,4
Hannover	108	Klagenfurt	13	68,4	Regensburg	14	93,3	Luzern	4	57,1	Jena	5	38,5
Berlin	96	Lübeck	14	66,7	Freiburg	12	92,3	Wien	22	51,2	Gießen	3	30
München	93	Ulm	28	63,6	Marburg	11	91,7	Lausanne	6	50	Kiel	8	29,6
Zürich	82	Mannheim	15	62,5	Dresden	9	90	Zürich	32	38,6	Tübingen	7	29,2
Innsbruck	70	Köln	75	59,1	Mannheim	13	86,7	Bern	8	38,1	Graz	6	28,6
Basel	53	Aachen	20	58,8	Rostock	5	83,3	Heidelberg	9	37,5	Essen	5	27,8
Göttingen	50	Bonn	23	57,5	Ulm	23	82,1	St. Augustin	3	37,5	Frankfurt	4	26,7
Kiel	48	Göttingen	30	55,6	Düsseldorf	8	81,8	Leipzig	7	33,3	Würzburg	5	26,3

Den höchsten Anteil der Publikationen mit ausschließlich Kollaborationspartnerschaften innerhalb des D‑A-CH-Raums hatten Publikationen aus Homburg, Klagenfurt (je 100 %) und Regensburg (93,3 %). Den höchsten Anteil der Publikationen mit ausschließlich Kollaborationspartnerschaften außerhalb des D‑A-CH-Raums hatten Publikationen aus Basel (64,1 %), Genf (60,0 %) und Luzern (57,1 %, Tab. 1).

**Abb. 5:**
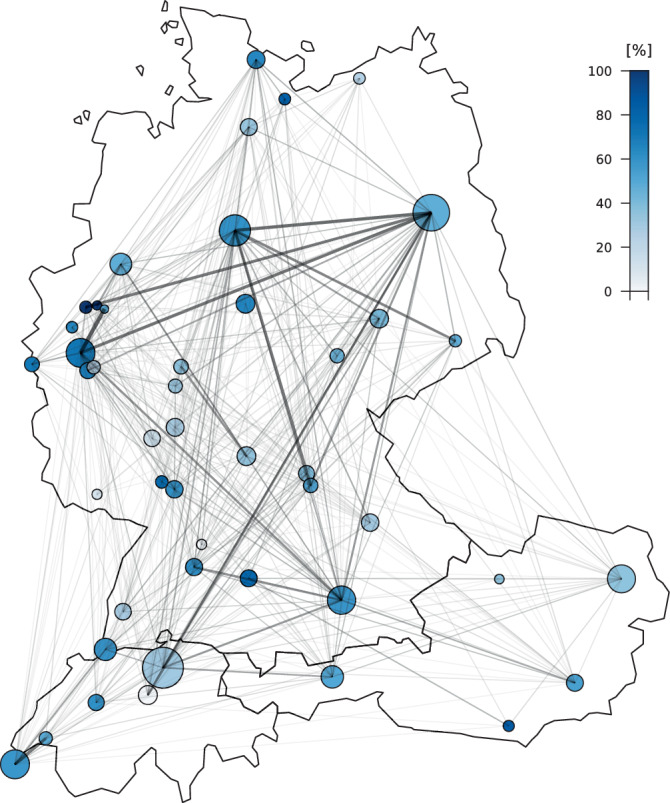
Aktivität (*Durchmesser*) und Vernetzung (*Farbe*) bei kinderanästhesiologischen Publikationen aus Deutschland, Österreich und Schweiz im Zeitraum 2001–2020. Je *dicker eine Verbindungslinie* zwischen 2 Städten ist, desto öfter kollaborierten Institutionen aus diesen. Angezeigt werden jene 46 Städte, aus denen von 2001 bis 2020 mindestens 10 Publikationen veröffentlicht wurden

Die drei häufigsten Kollaborationen waren zwischen den Städten Köln und Witten (21), Berlin und Hannover (18) sowie Berlin und Köln (17) (Abb. 5).

Eine interaktive Kartenversion ist online unter https://www.kinderanaesthesie-talk.de/szientometrie verfügbar.

### Publikationsorgane

Während Publikationen aus Deutschland am häufigsten in der Fachzeitschrift *Die Anaesthesiologie* erschienen (*n* = 154), wurden Publikationen aus Österreich und der Schweiz am häufigsten in *Pediatric Anesthesia* veröffentlicht (*n* = 39 bzw. *n* = 99, Tab. 2).

**Tab. 2:** Die 10 häufigsten Fachzeitschriften mit kinderanästhesiologischen Publikationen aus je Deutschland, Österreich und der Schweiz von 2001 bis 2020

Deutschland	*n*	Österreich	*n*	Schweiz	*n*
Die Anaesthesiologie	154	Pediatric Anesthesia	39	Pediatric Anesthesia	99
Pediatric Anesthesia	137	British Journal of Anaesthesia	22	Die Anaesthesiologie	45
Anästhesiologie & Intensivmedizin	107	Die Anaesthesiologie	17	British Journal of Anaesthesia	37
Anästhesiologie, Intensivmedizin, Notfallmedizin, Schmerztherapie	105	Anesthesia and Analgesia	15	Acta Anaesthesiologica Scandinavica	22
Notfall + Rettungsmedizin	45	Wiener klinische Wochenschrift	10	Anaesthesia	20
Anesthesia and Analgesia	37	Schmerz	9	European Journal of Anaesthesiology	17
European Journal of Anaesthesiology	32	Resuscitation	7	Anesthesia and Analgesia	16
British Journal of Anaesthesia	30	Anaesthesia	5	Current Opinion in Anaesthesiology	14
Monatsschrift Kinderheilkunde	29	Anästhesiologie, Intensivmedizin, Notfallmedizin, Schmerztherapie	5	Anaesthesiology	13
Notarzt	28	Pediatric Critical Care Medicine	5	Notfall + Rettungsmedizin	10

## Diskussion

Die Publikationsaktivität in der Kinderanästhesiologie in Deutschland, Österreich und der Schweiz nahm zwischen 2001 und 2020 jährlich um 3 % zu, wobei besonders Kollaborationen von wachsender Bedeutung sind. Dies spiegelt sich sowohl in einer mehr als doppelt so hohen Zunahme der Publikationen mit mehr als 2 kollaborierenden Institutionen als auch in der steigenden Anzahl an Co-Autorenschaften bei Publikationen mit Korrespondenz außerhalb von D‑A-CH-Raum wider. Außerdem kollaborieren die D‑A-CH-Staaten sehr stark miteinander.

Wissenschaftliches Arbeiten wird durch diverse rechtliche und organisatorische Anforderungen sowie Ressourcenknappheit zunehmend komplexer und aufwendiger [1, 13, 15, 18]. Daher ist ein hohes Maß an Expertise und Fachwissen erforderlich, um den ansteigenden Anforderungen mehr und mehr gerecht werden zu können. Eine zunehmende Vernetzung und Bündelung von Ressourcen eröffnet die Möglichkeit, bestehende Evidenzlücken gezielt(er) zu schließen [13] und besonders „schwierige Probleme“ gemeinsam angehen zu können [1]. Dies wird am besten durch Kollaborationen erreicht, was Adams als Grundpfeiler der aktuellen Forschungsepoche tituliert [1]. Besonders über Institutionsgrenzen hinaus haben Kollaborationen eine steigende Bedeutung: Sie bündeln Expertise und Ressourcen, ermöglichen größere Fallzahlen, erzeugen eine größere Resonanz, bringen „frische“ Ideen in bestehende Teams und tragen zur Dissemination von Fachwissen bei [3, 12, 19].

Kollaborationen mit all den genannten Aspekten sind daher für die Kinderanästhesiologie wesentlich, wie rezente Studien wie APRICOT oder NECTARINE, woran Wissenschaftler*innen aus 18 bzw. 30 Staaten mitwirkten, durch ihren Impact praxisrelevant darlegen. Es verwundert demnach nicht, dass der Anteil der durch Kollaborationen entstandenen, kinderanästhesiologischen Publikationen aus dem D‑A-CH-Raum überproportional angestiegen ist und knapp die Hälfte aller Publikationen mindestens eine Kollaboration aufweist. Die Zusammenarbeit findet überwiegend zwischen Institutionen innerhalb des D‑A-CH-Raums statt, was sicherlich durch die geografische und sprachliche Nähe begünstigt ist. Auffallend ist dabei ein deutlich höherer Anteil der internationalen Kollaborationen von Schweizer Institutionen als von Deutschen oder Österreichischen. Internationalität scheint in der schweizerischen Kinderanästhesiologie-Forschung eine größere Rolle zu spielen, womöglich aufgrund der geringeren Zahl der wissenschaftlich tätigen Institutionen. Dies würde zwar auf Österreich auch zutreffen, jedoch ist dort die Publikationsaktivität generell geringer und die Vernetzung mit Deutschland stärker. Österreichische und schweizerische Institutionen publizieren, relativ gesehen, häufiger in internationalen Fachzeitschriften wie *Pediatric Anesthesia.* Deutsche Institutionen hingegen wählen am häufigsten deutschsprachige Fachzeitschriften – allen voran *Die Anaesthesiologie*, welche aber auch für Österreich und Schweiz eine häufig gewählte Fachzeitschrift ist.

Trotz Zunahme der Publikationsaktivität ist das jährliche Wachstum im D‑A-CH-Raum etwas geringer als das Weltweite, das im selben 20-Jahres-Zeitraum 7,6 % betrug [9] und v. a. auf die teilweise hohen Wachstumsraten einiger Länder wie China und Indien zurückzuführen ist. Dies spiegelt sich auch in einem seit 2016 erkennbaren Abrutsch der D‑A-CH-Staaten im länderspezifischen Ranking der Korrespondenzautorenschaften wider [9] und ist in ähnlicher Weise auch auf dem Gebiet der gesamten Anästhesiologie zu beobachten [2]. Dennoch gehören D‑A-CH zu den in den letzten beiden Dekaden am häufigsten kinderanästhesiologisch publizierenden Staaten.

Bezüglich der Verteilung fällt die Ähnlichkeit zu einer generalisierten Pareto-Verteilung auf [17], da ca. 80 % der Institutionen weniger als 20 Publikationen hatten. Sehr wenige Institutionen sind also für einen Großteil der Publikationen verantwortlich, was als Konzentrierung der Korrespondenzen gesehen werden kann und damit eine Spezialisierung in der kinderanästhesiologischen Publikationslandschaft indiziert. Mehr als die Hälfte der wissenschaftlich aktiven Institutionen sind zwar an Publikationen beteiligt, waren aber selbst nie als Korrespondenz benannt, also vermutlich nicht federführend. Dies spiegelt sich auch anhand der steigenden Anzahl an Co-Autorenschaften pro Publikation sowohl in unseren Daten und auch in der Anästhesiologie gesamt wider [16]. Allerdings steigt nicht nur die Anzahl an Autorenschaften, sondern auch die Anzahl an verschiedenen, an einer Publikation beteiligten Institutionen und damit der Grad an Vernetzung. Im Gegenzug sinkt der Anteil jener Publikationen, die von einer einzigen Institution ohne jegliche Kollaborationen ausgehen. Dies zeigt einen eindeutigen Trend zur Vernetzung auf breiterer Basis, könnte aber auch ein Indikator für eine Zunahme der wissenschaftlichen Komplexität sein.

Bezüglich einer weiteren Vernetzung auf Ebene des D‑A-CH-Raums kommt dem Wissenschaftlichen Arbeitskreis Kinderanästhesie der DGAI sicherlich eine tragende Rolle bei der Ermöglichung von Kollaborationen zu. Auf europäischer Ebene wurde bei der European Society of Anaesthesiology and Intensive Care (ESAIC) das *Paediatric Anaesthesia Research Network* (ESAIC PARNet) mit dem Ziel eingerichtet, interessierten Wissenschaftler*innen Kollaborationen unter dem Schirm dieser Institution zu ermöglichen.

Szientometrische Analysen lassen jedoch einige Aspekte offen: Hinter bloßen Zahlen stecken oft Parameter, die nicht final beantwortet werden können [11, 14]. Sind alle Autorenschaften gerechtfertigt? Haben alle Beteiligten gleich viel beigetragen? Ist viel gut oder ist weniger mehr? Welche Ressourcen stecken hinter einer Publikation? Die berichteten Zahlen sind zumindest ein Indikator für die Ableitung eines longitudinalen Trends, da die Verwendung eines geeigneten Nenners die Verzerrung reduziert [10].

### Limitationen

Wir sind uns mehrerer Limitationen bewusst: Erstens kann das Abfrageschema zu einer Überschätzung der Publikationsaktivität geführt haben, da die Kombination aus kinderbezogenen Themen und Zugehörigkeit zu einer anästhesiologischen Institution nicht zwangsläufig ausschließlich kinderanästhesiologisch spezifische Publikationen erfasst. Dies gilt z. B. für kinderintensivmedizinische Publikationen. Da es im D‑A-CH-Raum (und auch außerhalb) nur wenige eigenständige Abteilungen für Kinderanästhesiologie gibt, steht kein adäquateres Abfrageschema zur Vermeidung falsch-positiver Erfassungen zur Verfügung [9]. Zumindest waren bei den erfassten Publikationen anästhesiologisch affiliierte Personen Teil des Teams. Zudem lässt die Verteilung auf die identifizierten Fachzeitschriften auf eine hohe Sensitivität für kinderanästhesiologisch relevante Themen schließen. Zweitens wurden nur vollständig genannte Autorenschaften gezählt; die noch nicht lange bestehenden „collaborative authorships“ wurden durch das Abfrageschema nicht erfasst. Dies könnte die Anzahl der Kollaborationen sogar noch unterschätzt haben. Drittens ist die Unterscheidung zwischen Institution und Stadt nicht immer scharf trennbar, da es manche Institutionen gibt, die einer anderen Stadt zugeordnet sind und daher zu Doppelt- oder Falschnennungen führen können. Wir haben uns dabei auf die jeweils angegebene Zugehörigkeit gestützt. Viertens sind Analysen über das *Affiliation field *auf eine korrekte Angabe der Zugehörigkeitsfelder durch Wissenschaftler*innen sowie konsekutiv Fachzeitschriften und Datenbanken angewiesen. Fehlerhafte oder unzureichende Eingaben, wie sie v. a. bei PubMed immer wieder vorkommen [15], können bei Analysen nicht korrigiert werden.

## Fazit für die Praxis

- Die Anzahl der Publikationen im Bereich Kinderanästhesiologie aus dem D‑A-CH-Raum nahm zwischen 2001 und 2020 zu.
- Kollaborationen spielten dabei eine zunehmend wichtigere Rolle, was durch die überproportionale Steigerung an Publikationen mit mehreren Institutionen angezeigt wird.
- Vorwiegend wurde innerhalb des D‑A-CH-Raums kooperiert; nur in der Schweiz war der Grad der internationalen Kollaboration deutlich höher.
- Der Grad der Vernetzung bei kinderanästhesiologischen Publikationen nahm ebenfalls zu, zeigte jedoch eine Konzentrierung an federführenden Institutionen.
- Es bleibt zu wünschen, dass dies in einer Verbesserung der Evidenzlage in der Kinderanästhesie resultiert.
